# Genetic Variants in Nitric Oxide Synthase Genes and the Risk of Male Infertility in a Chinese Population: A Case-Control Study

**DOI:** 10.1371/journal.pone.0115190

**Published:** 2014-12-17

**Authors:** Lifeng Yan, Wenhui Guo, Shengmin Wu, Jining Liu, Shenghu Zhang, Lili Shi, Guixiang Ji, Aihua Gu

**Affiliations:** 1 State Key Laboratory of Reproductive Medicine, Institute of Toxicology/Key Laboratory of Modern Toxicology of Ministry of Education, School of Public Health, Nanjing Medical University, Nanjing, China; 2 Nanjing Institute of Environmental Sciences/Key Laboratory of Pesticide Environmental Assessment and Pollution Control, Ministry of Environmental Protection, Nanjing, China; MOE Key Laboratory of Environment and Health, School of Public Health, Tongji Medical College, Huazhong University of Science and Technology, China

## Abstract

**Background:**

In recent years, oxidative stress has been studied extensively as a main contributing factor to male infertility. Nitric Oxide, a highly reactive free radical gas, is potentially detrimental to sperm function and sperm DNA integrity at high levels. Thus, the aim of this study was to investigate the associations between five polymorphisms in nitric oxide synthase genes (NOSs) and the risk of male infertility and sperm DNA damage as well.

**Methods:**

Genotypes were determined by the OpenArray platform. Sperm DNA fragmentation was detected using the Tdt-mediated dUTP nick-end labeling assay, and the level of 8-hydroxydeoxyguanosine (8-OHdG) in sperm DNA was measured using immunofluorescence. The adjusted odds ratio (OR) and 95% confidence interval (CI) were estimated using unconditional logistic regression.

**Results:**

Our results revealed a statistically significant difference between the cases and controls in both genotypic distribution (*P*<0.001) and allelic frequency (*P* = 0.021) only for the *NOS3* rs1799983 SNP. Multivariate logistic regression analyses revealed that rs1799983 was associated with a borderline significantly increased risk of male infertility (GT vs. GG: adjusted OR = 1.30, 95% CI: 1.00–1.70; GT+TT vs. GG: adjusted OR = 1.34, 95% CI: 1.03–1.74; *P*_trend_ = 0.020). Moreover, *NOS3* rs1799983 was positively associated with higher levels of sperm DNA fragmentation (β = 0.223, *P* = 0.044). However, the other 4 polymorphisms (*NOS1* rs2682826, *NOS1* rs1047735, *NOS2* rs2297518, and *NOS2* rs10459953) were not found to have any apparent relationships with male infertility risk.

**Conclusions:**

Of five *NOS* gene polymorphisms investigated in the present study, we found *NOS3* rs1799983 might cause oxidative sperm DNA damage, thereby contributing to male infertility.

## Introduction

Infertility is a common reproductive disorder that affects approximately one in six couples globally and male factors account for 40 to 50% of all infertile cases [1], [2]. Though the aetiologies are still poorly understood, oxidative stress has been shown to be associated with impaired sperm motility and male infertility [3], [4].

Nitric Oxide (NO) is a highly reactive free radical gas and can act as an ‘acceptor’ and inactivate superoxide (O_2_^−^) [5]. NO modulates sexual and reproductive functions has been previously identified in mammalian species [6], [7]. NO is detectable in seminal plasma and NO concentration in seminal plasma of some infertile males has been demonstrated to be significantly higher than the healthy males [8]. NO is reported to be a novel mediator of sperm function [9], and the effects on sperm are dose-dependent: at physiological concentrations, NO has positive effects on sperm capacitation and acrosome reaction [10], [11], while at excessive concentrations, NO has negative effects on sperm motility and viability [12], [13].

NO are generated from the oxidation of L-arginine to L-citrulline by nitric oxide synthase (NOS) enzymes [14]. In mammals, 3 distinct genes encode for NOS isozymes, namely, neuronal NOS (*nNOS* or *NOS1*, 150 kd on chromosome 12[12q24.2–q24.31]), inducible NOS (*iNOS* or *NOS2*, 130 kd on chromosome 17 [17q11.2–q12]), and endothelial NOS (*eNOS* or *NOS3*, 135 kd on chromosome 7 [7q35–q36]) [15]. Genetic polymorphisms of *NOS* have been studied extensively in susceptibility to schizophrenia, schizoaffective disorder, asthma, Parkinson's disease, hepatitis, diabetic retinopathy, rheumatoid arthritis, cancer,cardiovascular,and inflammatory diseases [16]–[18]. Recently, the associations between *NOS3* gene polymorphisms and male infertility were also reported [19]–[22]. NOS3-786CC, 894TT and 4aa genotype were significantly more frequent in infertile subjects, and 786C, 894T, and 4a alleles contributed to poor semen parameters,suggesting a significant relationship between NOS genotypes and the phenotype of male infertility [19], [21]. Previous studies also showed that NOS was involved in sperm motility, capacitation, and acrosome reaction [9], and NOS activity was found in Leydig and Sertoli cells of the testis, penile corpora cavernosa, and spermatozoa [23]–[25].

In view of the importance of NO and NOS in male reproductive function, we hypothesized that the *NOS* gene polymorphisms may represent a common genetic factor towards the development of male infertility. Thus, the aim of the present study was to investigate the associations between five polymorphisms in *NOS* genes (*NOS1* rs2682826, *NOS1* rs1047735, *NOS2* rs2297518, and *NOS2* rs10459953, and *NOS3* rs1799983) and male infertility risk and also sperm DNA damage in a Chinese population.

## Methods and Materials

### Subjects and sample collection

The study was approved by the Ethics Review Board of Nanjing Medical University. All the studies involving human subjects were conducted in full compliance with government policies and the Declaration of Helsinki. A total of 1657 infertile patients diagnosed with unexplained male factor infertility were drawn from the Center of Clinical Reproductive Medicine between April 2005 and March 2009 (NJMU Infertility Study). All patients underwent at least two semen analyses, and those with a history of orchitis, obstruction of the vas deferens, chromosomal abnormalities, or microdeletions of the azoospermia factor region on the Y chromosome were excluded. In the final analysis, 580 idiopathic infertility patients aged 24 to 42 years were included. The control group consisted of 580 fertile men ranging from 25 to 40 years old who had fathered at least one child without assisted reproductive technologies from the early pregnancy registry at the same hospitals where the patients were recruited. All participants were ethnic Han Chinese and completed a written informed consent and a questionnaire including detailed information, such as age, cigarette smoking, alcohol intake, tea and vitamin consumption, and abstinence time. Each subject donated 5 ml of blood for genomic DNA extraction, and an ejaculate for routine semen analysis, the assessment of sperm DNA fragmentation, and the assessment of sperm 8-OHdG. The semen analysis for sperm concentration, and motility was performed following the World Health Organization criteria [26].

### SNP selection and genotyping

Through an extensive mining of the databases of the International HapMap Project and dbSNP, we identified five potential functional polymorphisms in *NOS* genes. All of these SNPs have a reported minor allele frequency of ≥0.05 in the general Han Chinese population.

Briefly, DNA was extracted from leukocyte pellets of the venous blood by phenol-chloroform extraction with proteinase K digestion, and frozen until use. Genotype analysis was done using the OpenArray platform (Applied Biosystems, Foster City, CA, USA), which employs a chip-based Taq-Man genotyping technology. Genotyping was conducted according to the manufacturer's standard protocols, and genotype calls were made by OpenArray SNP Genotyping Analysis Software version 1.0.3. For quality control, 10% of the samples were randomly genotyped twice by different individuals, and the reproducibility was 100%. To confirm the genotyping results, selected PCR-amplified DNA samples (n = 2, for each genotype) were examined by DNA sequencing and the results were also consistent.

### DNA fragmentation analysis

A detailed protocol of the Terminal-deoxynucleoitidyl Transferase Mediated Nick End Labeling (TUNEL) assay for human sperm has been described previously [27]. We used flow cytometry to detect TUNEL staining of sperm from patients with sperm concentrations >5×10^6^/ml. This assay has been shown to be a feasible and sensitive way to detect DNA fragmentation [28]. TUNEL labeling was carried out using a Cell Death Detection kit (APO-DIRECT kit; BD Biosciences PharMingen) according to the manufacturer's instructions. Briefly, semen samples, frozen at −70°C, were thawed in a 37°C water bath and immediately diluted with buffer (0.15 M NaCl, 0.01 M Tris, 0.001 M EDTA, pH 7.4) to obtain a sperm concentration of 1−2×10^6^/ml. Washed sperm were resuspended in 2% paraformaldehyde for 30 min at room temperature. After rinsing in PBS, samples were resuspended in permeabilization solution (0.2% Triton X-100, 0.1% sodium citrate) for 10 min on wet ice. TUNEL reagent (50 µl) was added to each sample. For each batch, a negative control lacking the terminal deoxynucleotidyl transferase and a positive control treated with DNase I were included to ensure assay specificity. After incubation for 1 h at 37°C, samples were analyzed immediately by flow cytometry (FACSCalibur; BD Biosciences Pharmingen). Flow during the analysis was controlled at approximately 500 spermatozoa/sec, and 10,000 cells were analyzed for each sample. The percentage of FITC-positive cells (FL1 channel) was calculated as the percentage of cells with a fluorescence intensity exceeding the threshold obtained with the negative control.

### Sperm 8-OHdG determination

Sperm 8-hydroxydeoxyguanosine (8-OHdG), as a biomarker of sperm oxidative damage, was measured by a direct immunofluorescence method using a specific antibody (Biotrin OxyDNA Test kit; Biotrin International Ltd.) conjugated to FITC. The FITC fluorescence can then be quantified using the flow cytometer (FACSCalibur; BD Biosciences Pharmingen) as described previously [29].

Briefly, washed sperm were resuspended in 4% paraformaldehyde at 8°C for 15 min. After rinsing in PBS, samples were resuspended in permeabilization solution (0.2% Triton X-100, 0.1% sodium citrate) for 10 minutes on wet ice and washed in wash solution (Biotrin Oxy-DNA Test kit). Before incubation for 1 h at 37°C, 50 µl of freshly prepared blocking solution (Biotrin OxyDNA Test kit) was added to each sample to block the nonspecific binding sites. The purified anti-8-OHdG antibody was then added at a 1∶50 dilution to the fixed cells in enough wash solution to achieve a final volume of 100 µl, before incubation for 1 h. After washed twice, the cells were resuspended in 1 ml of PBS, and transferred to 5-ml fluorescence-activated cell sorter tubes for flow cytometric analysis. For each batch, a negative control with mouse IgG instead of the anti-8-OHdG antibody and a positive control treated with 2 mM H_2_O_2_ and 1 mM FeCl_2_·4H_2_O were included to ensure assay specificity.

### Statistical analyses

All the statistical analyses were performed using the STATA 11.0 (STATA Corp, College Station, Texas). All *P*-values were two-sided and the significance level was set at *P*<0.05. Differences in selected demographic variables as well as smoking and alcohol status between the cases and the controls were evaluated by the χ^2^ test. The Student's t test was used to evaluate continuous variables, including age and pack-years of cigarette smoking. A goodness-of-fit χ^2^ test was used to determine the Hardy-Weinberg equilibrium of the observed genotype frequencies. Unconditional multivariate logistic regression analysis was employed to examine the association between genetic polymorphisms and male infertility risk by estimating odds ratios (ORs) and 95% confidence intervals (95% CI). Genotypes were categorized into three groups (major allele homozygous, heterozygous, and homozygous variant). Age, cigarette smoking, alcohol intake were considered as covariates. Two-sided χ^2^ test were performed to estimate the difference in frequency distribution of genotypes and alleles between cases and controls. Sperm DNA fragmentation and sperm 8-OHdG were normalized by natural logarithm (ln) transformation. Linear regression models were used to estimate the association with ln-transformed sperm fragmentation and ln-transformed 8-OHdG values for each SNP independently. Models were adjusted age, smoking status, drinking status and abstinence time.

## Results

### Characteristics of the study population

The selected characteristics of the 580 cases and 580 controls included in this study are summarized in Table 1. The characteristics of the subjects have been described previously [30]. In summary, case patients and control subjects were adequately matched on age, drinking status, Body Mass Index (BMI) and abstinence time (*P*>0.05). However, there were more smokers in the case group compared with the control group (*P* = 0.046). Among smokers, cases also reported significantly greater cigarette consumption than controls, as assessed by the mean number of pack-years (*P* = 0.019). Moreover, there was a significant difference between cases and controls with respect to semen concentration (cases versus controls: 73.6±49.37×10^6^/ml versus 115.2±76.58×10^6^/m, *P*<0.001) and sperm motility (37.9±13.49% versus 65.3±12.52%, *P*<0.001), but not semen volume (*P* = 0.809).

**Table 1:** Distribution of selected characteristics between cases and fertile controls.

Variables	Cases *N* = 580 (%)	Controls *N* = 580 (%)	*P* b
Age			
<30	326 (56.3)	299 (51.5)	0.238
≥30	254 (43.7)	281 (48.5)	
Age (mean ± SD)	28.2±3.3	28.1±3.2	0.600
Smoking status			
Never	272 (46.9)	306 (52.8)	**0.046**
Ever	308 (53.1)	274 (47.2)	
Pack-years (mean ± SD)^a^	4.7±4.4	4.1±4.3	**0.019**
Drinking status			
Never	500 (86.2)	504 (86.8)	0.731
Ever	80 (13.8)	76 (13.1)	
BMI (kg/m^2^)			
<25	423	413	0.246
25–29.9	149	153	
≥30	8	14	
BMI (mean ± SD) (kg/m^2^)	23.3±0.1	23.4±0.1	0.825
Abstinence time (days)			
Semen parameters (mean ± SD)			
Concentration (×10^6^/ml)	73.6±49.37	115.2±76.58	**<0.001**
Motility (%)	37.9±13.49	65.3±12.52	**<0.001**
Volume (ml)	2.78±1.44	2.80±1.37	0.809
Sperm DNA fragmentation (%)	20.98±15.30	n. d.	

a Among ever smokers.

b *P* values were derived from the χ^2^ test for categorical variables (Age, BMI, smoking and drinking status) and t test for continuous variables (age, pack-years and BMI).

n. d.: not detected.

### Individual single nucleotide polymorphism and susceptibility to male infertility

The position and minor allele frequency of the five functional SNPs found in the Chinese population in the HapMap database are presented in Table 2. All tested genotypes were in Hardy-Weinberg equilibrium in control groups (*P*>0.05). The main effect models for all the genotypes are presented in Table 3. A statistically significant difference was observed between the cases and controls in both genotypic distribution (*P*<0.001) and allelic frequency (*P* = 0.021) only for the rs1799983 SNP. Multivariate logistic regression analyses revealed that rs1799983 GT heterozygotes was associated with a borderline significantly increased risk of male infertility (adjusted OR = 1.30, 95% CI: 1.00–1.70). In the dominant model, combined rs1799983 genotypes (GT/TT) were associated with a significantly 34% increased risk of male infertility (adjusted OR = 1.34, 95% CI: 1.03–1.74). Trend χ^2^ test showed that the male infertility risk was significantly increased in a dose-dependent manner (*P*_trend_ = 0.020). However, we did not found other four polymorphisms (*NOS1* rs2682826, *NOS1* rs1047735, *NOS2* rs2297518, and *NOS2* rs10459953) have any apparent relationship with risk of male infertility.

**Table 2:** SNPs information.

Genotyped SNPs	Location/or Amino acid change	MAF for Chinese in database*	*P* value for HWE test	% Genotyping rate
*NOS1*: rs2682826 C>T	3' UTR	0.256	0.086	99.1
*NOS1*: rs1047735 T>C	nsSNP/Q902H	0.488	0.432	98.9
*NOS2*: rs2297518 G>A	nsSNP/L608S	0.175	0.327	98.4
*NOS2*: rs10459953 G>C	Promoter	0.489	0.744	99.1
*NOS3*: rs1799983 G>T	nsSNP/D298E	0.111	0.503	98.9

Abbreviations: MAF, minor allele frequency; HWE, Hardy-Weinberg equilibrium.

*Minor allele frequency in the Chinese (CHB, Han Chinese in Beijing, China) population, as reported in dbSNP database.

**Table 3:** Associations between *NOS* genotypes and the risk of male infertility.

Genotype	Cases *N* = 580 (%)	Controls *N* = 580 (%)	*P*	Adjusted OR (95% CI)^a^
rs2682826 (C>T)	575	575		
CC	318	292	0.050 b	1.00
CT	209	247		0.78 (0.61–1.02)
TT	48	36	0.511^c^	1.22 (0.77–1.93)
T allele frequency	0.265	0.277	0.376 d	
CT/TT	257	283		0.90 (0.85–1.05)
rs1047735 (T>C)	576	571		
TT	144	153	0.252 b	1.00
TC	306	276		1.21 (0.91–1.60)
CC	126	142	0.772 c	0.97 (0.70–1.35)
C allele frequency	0.484	0.490	0.774 d	
TC/CC	432	418		1.13 (0.87–1.47)
rs2297518 (G>A)	571	570		
GG	392	385	0.917 b	1.00
GA	166	171		0.95 (0.74–1.23)
AA	13	14	0.677 c	0.91 (0.42–1.96)
A allele frequency	0.168	0.175	0.683 d	
GA/AA	179	185		0.97 (0.75–1.24)
rs10459953 (G>C)	578	573		
GG	169	184	0.527 b	1.00
GC	287	278		1.14 (0.87–1.48)
CC	122	111	0.268 c	1.21 (0.87–1.69)
C allele frequency	0.459	0.436	0.266 d	
GC/CC	409	389		1.17 (0.91–1.51)
rs1799983 (G>T)	578	569		
GG	410	435	**<0.001** b	1.00
GT	154	127		**1.30 (1.00–1.70)**
TT	14	7	**0.020** c	2.14 (0.85–5.35)
T allele frequency	0.157	0.124	**0.021** d	
GT/TT	168	134		**1.34 (1.03–1.74)**

a Adjusted by age, pack-years of smoking, and alcohol use.

b Two-sided χ^2^ test for difference in frequency distribution of genotypes between cases and controls.

c *P* trend for genotypes between cases and controls.

d Two-sided χ^2^ test for difference in frequency distribution of alleles between cases and controls.

Data in *boldface* represent *P*<0.05.

### Effects of *NOS* SNPs on sperm DNA damage levels

**Figure 1:**
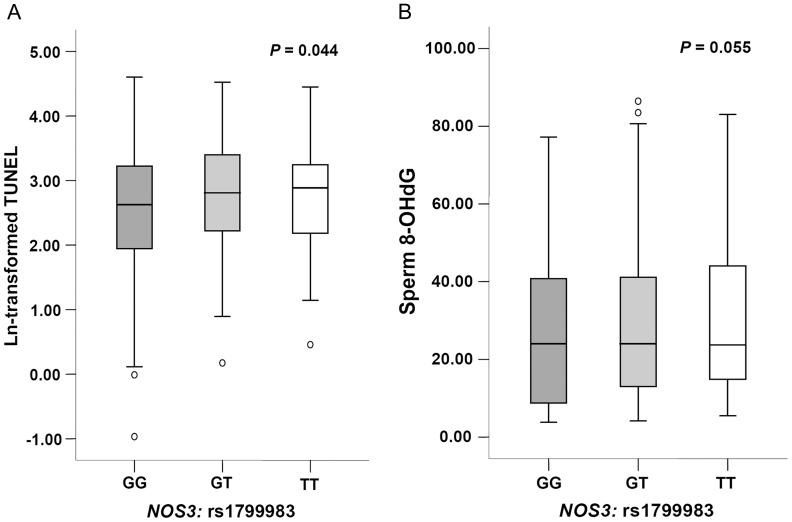
Box-and-whisker plots for sperm DNA damage levels in study subjects. The boxes represent the 25th and 75th percentiles; whiskers are lines extending from each end of the box covering the extent of the data on 1.5× inter-quartile range. The median value is denoted as the line that bisects the boxes. Circles represent the outlier values.

Considering the fact that NO induced oxidative stress can cause sperm DNA damage, we further evaluated the effects of these functional genetic variants in *NOS* genes on sperm DNA integrity and the levels of 8-OHdG. To determine whether the results of the TUNEL analyses were profoundly influenced by cryopreservation in our study, ten semen samples were pre-treated with or without cryopreservation prior to TUNEL analyses. As showed in S1 Table, modest but significant elevated levels of sperm DNA fragmentation induced by cryopreservation (*P* = 0.001). However, all the semen samples undergo the same cryopreservation process, thus we believe that the cryopreservation, if any, is unlikely to be substantial. The analyses for associations between polymorphisms and sperm DNA fragmentation and 8-OHdG levels are summarized in Table 4. The direction of the regression coefficient (β) represents the effect of each minor allele increasing (+) or decreasing (−) the values of sperm DNA fragmentation or 8-OHdG. We observed that the *NOS3* rs1799983 variants were positively associated with higher levels of sperm DNA fragmentation (β = 0.223, *P* = 0.044) (Fig. 1).

**Table 4:** Effects of *NOS* SNPs on sperm DNA damage levels.

Genotyped SNPs	Sperm DNA fragmentation			Sperm 8-OHdG		
	Beta^a^	SE	*P*	Beta^a^	SE	*P*
*NOS1*: rs2682826	−0.171	0.104	0.093	−3.490	4.126	0.399
*NOS1*: rs1047735	0.051	0.088	0.558	3.528	4.112	0.392
*NOS2*: rs2297518	0.164	0.089	0.065	2.180	4.050	0.591
*NOS2*: rs10459953	0.145	0.094	0.201	0.635	4.601	0.890
*NOS3*: rs1799983	0.223	0.110	**0.044**	7.952	4.118	0.055

Beta, regression coefficient; SE, standard error.

a All analysis were done using linear regression models, adjusted for age, smoking status, drinking status and abstinence time.

Data in *boldface* represent *P*<0.05.

## Discussion

Infertility has multi-factorial etiologies, including genetic and environmental risk factors. NO, as one of the most potent free radicals of nitrogen, reacts rapidly with superoxide to form highly toxic peroxynitrite, both of which have the ability to damage DNA directly [31]. Sperm DNA integrity is essential for the accurate transmission of genetic information, so sperm DNA damage could result in male infertility regardless of sperm number, motility and morphology [32], [33]. In addition, DNA damage in human spermatozoa is associated with low fertilisation rates, impaired preimplantation development, increased abortion and an elevated incidence of disease in the offspring (e.g. childhood cancer) [34], [35]. Thus, this study was performed to investigate the associations between five polymorphisms in NOS genes with sperm DNA damage, and male infertility risk in a Chinese population.

Previous studies have examined the relationship of *NOS3* gene polymorphisms and male infertility, but the results were conflicting. Yun *et al*. reported no significant associations between *NOS3* 786T>C, 4a4b, and 894G>T of the control and infertile group in a Korean population, nevertheless, 4a4b polymorphism was significantly associated with sperm morphology [22]. Similarly, Bianco *et al*. found the three genetic variations of *NOS3* were not associated with male infertility in a Brazilian population [20]. However, Safarinejad *et al*. found that the *NOS3*−786CC, 894TT and 4aa genotype were significantly more frequent in infertile subjects, and 786C, 894T, and 4a alleles was associated with an increased risk of poor semen parameters [19]. Buldreghini *et al.* showed T allele of *NOS3* G894T polymorphism contributed to poor sperm motility [21]. In the present case-control study, our results revealed that *NOS3* rs1799983 (G894T) was associated with a borderline significantly increased risk of male infertility (GT *vs* GG: adjusted OR = 1.30, 95% CI: 1.00–1.70; GT+TT *vs* GG: adjusted OR = 1.34, 95% CI: 1.03–1.74; *P*_trend_ = 0.020).

The *NOS3* rs1799983 (G894T) is located in exon 7, where glutamic acid is substituted at codon 298 for aspartic acid. Computational analysis has also revealed that this mutation might affect the protein-protein interactions and localization of the NOS3 protein, which might also affect the protein function and explain the enhanced disease risk associated with the presence of G894T polymorphism in the NOS3 protein [36]. Additionally, T allele was demonstrated to generate protein products with different susceptibility to cleavage, suggesting that this polymorphism has a functional effect on the NOS3 protein [37]. Furthermore, the 894T allele is associated with high plasma NO levels in healthy people [38] and high protein levels of NOS3 [39].

In this study, we also demonstrated a significant positive association of *NOS3* rs1799983 variants with higher levels of sperm DNA fragmentation (β = 0.223, *P* = 0.044) in infertile men. NO concentration in the seminal plasma of infertile males was evaluated to be significantly higher than fertile males and NO concentration was positively associated with sperm DNA damage in infertile males [40]. Though the mechanism responsible for the relationship between the *NOS3* polymorphism and risk of infertility is not known at present, we hypothesis that *NOS3* rs1799983 variants may have higher NOS3 levels and activity, resulting in a high concentration of NO; a high NO concentration may cause sperm DNA damage, thereby contributing to male infertility.

Several strengths of our study should be acknowledged. 1) All tested SNPs were in Hardy-Weinberg equilibrium in controls; 2) All cases and controls were racially homogeneous (all Han-Chinese) and well matched with regard to age, cigarette smoking, and drinking status, which minimises potential confounding bias; 3) The genotyping method for the study was standardised, and quality control samples indicated high reproducibility of the genotyping results. However, some limitations might exist. 1) The moderate sample size might not be sufficient for us to adequately detect a small effect from very low-penetrance SNPs; 2) we were unable to provide neither seminal plasma levels of NO in subjects with different genotypes nor specific biological mechanism of how *NOS3* rs1799983 affect male infertility.

## Conclusions

In summary, the findings demonstrate that, in Chinese population studied, four polymorphisms of NOS genes, namely, *NOS1* rs2682826, *NOS1* rs1047735, *NOS2* rs2297518, and *NOS2* rs10459953 were not correlated with male infertility. But we showed that *NOS3* rs1799983 was significantly associated with risk of male infertility and sperm DNA damage. Our founding might be helpful in improving our understanding of the genetic susceptibility of sperm DNA integrity and in providing diagnostic implications for assisted reproduction success rates. Future large-scale studies with ethnically diverse populations and functional evaluations are needed to validate our findings.

## Supporting Information

S1 Table **Effect of cryopreservation on sperm DNA fragmentation.** Ten semen samples were pre-treated with or without cryopreservation prior to TUNEL analyses. T-test analysis showed a modest but significant elevated levels of sperm DNA fragmentation induced by cryopreservation (*P* = 0.001).
(DOC)
