# Supplementary material for: Genetic Variants in Nitric Oxide Synthase Genes and the Risk of Male Infertility in a Chinese Population: A Case-Control Study
Source: PLoS One. 2014 Dec 17;9(12):e115190. doi: 10.1371/journal.pone.0115190 (PMC4269448; doi:10.1371/journal.pone.0115190)
Supplement: S1 Table — Effect of cryopreservation on sperm DNA fragmentation. Ten semen samples were pre-treated with or without cryopreservation prior to TUNEL analyses. T-test analysis showed a modest but significant elevated levels of sperm DNA fragmentation induced by cryopreservation (P = 0.001). (DOC) [file pone.0115190.s001.doc]

Table S1. Effect of cryopreservation on sperm DNA fragmentation.

|  | Sperm DNA fragmentation | | | | | | | | | | |
| --- | --- | --- | --- | --- | --- | --- | --- | --- | --- | --- | --- |
| No. | 1 | 2 | 3 | 4 | 5 | 6 | 7 | 8 | 9 | 10 | mean±SE |
| cryopreservation | 35.8 | 24.8 | 15.2 | 26.7 | 35.8 | 43.7 | 32.5 | 19.2 | 27.7 | 17.8 | 27.9±2.9^*^ |
| Fresh sample | 32.5 | 23.9 | 15.1 | 23.3 | 31.7 | 38.3 | 31.5 | 17.1 | 25.5 | 16.0 | 25.5±2.5 |

Note: asterisk marks results of T-test for independent sample: ^*^*P* < 0.01.
